# Transient Abnormal Myelopoiesis and AML in Down Syndrome: an Update

**DOI:** 10.1007/s11899-016-0338-x

**Published:** 2016-08-10

**Authors:** Neha Bhatnagar, Laure Nizery, Oliver Tunstall, Paresh Vyas, Irene Roberts

**Affiliations:** 1 Children’s Hospital, John Radcliffe Hospital, Oxford University Hospitals NHS Foundation Trust, Oxford, OX3 9DU UK; 2 Paediatric Intensive Care Unit, Robert Debré Hospital, 48 Boulevard Sérurier, 75019 Paris, France; 3 Bristol Royal Hospital for Children, Paul O’Gorman Building, Upper Maudlin St, Bristol, BS2 8B UK; 4 Molecular Haematology Unit, Weatherall Institute of Molecular Medicine, University of Oxford, John Radcliffe Hospital, Oxford, OX3 9DS UK; 5 Department of Paediatrics, Children’s Hospital, University of Oxford, John Radcliffe Hospital, OX3 9DU Oxford, UK

**Keywords:** Transient abnormal myelopoiesis, Down syndrome, Acute leukaemia, Myeloproliferative disorders

## Abstract

Children with constitutional trisomy 21 (Down syndrome (DS)) have a unique predisposition to develop myeloid leukaemia of Down syndrome (ML-DS). This disorder is preceded by a transient neonatal preleukaemic syndrome, transient abnormal myelopoiesis (TAM). TAM and ML-DS are caused by co-operation between trisomy 21, which itself perturbs fetal haematopoiesis and acquired mutations in the key haematopoietic transcription factor gene *GATA1*. These mutations are found in almost one third of DS neonates and are frequently clinically and haematologcially ‘silent’. While the majority of cases of TAM undergo spontaneous remission, ∼10 % will progress to ML-DS by acquiring transforming mutations in additional oncogenes. Recent advances in the unique biological, cytogenetic and molecular characteristics of TAM and ML-DS are reviewed here.

## Introduction

Population studies show that children with Down syndrome due to constitutional trisomy 21 have a markedly increased risk of developing acute leukaemia compared with children without Down syndrome [1]. Both myeloid leukaemia, known as myeloid leukaemia of Down syndrome (ML-DS), and acute lymphoblastic leukaemia are increased by 150- and ∼30-fold, respectively [1, 2]. ML-DS has a distinct natural history and clinical and biological features (reviewed in [3, 4]). It virtually always develops before the age of 5 years, and the acute leukaemia is preceded by a clonal neonatal preleukaemic syndrome known as transient abnormal myelopoiesis (TAM) that is unique to Down syndrome [3, 4].

TAM is characterised by increased circulating blast cells that harbour acquired N-terminal truncating mutations in the key haematopoietic transcription factor gene *GATA1* [5–10]. Around 10–15 % of neonates with Down syndrome have a diagnosis of TAM with blasts >10 % and typical clinical features that require close monitoring in the neonatal period since the mortality rate may be up to 20 %. A further 10–15 % of neonates with Down syndrome have one or more acquired *GATA1* mutations in association with a low number of circulating blast cells (<10 %) and have clinically and haematologically silent disease (silent TAM) [11••]. In the majority of cases of TAM and silent TAM, the *GATA1* mutant clone goes into complete and permanent remission without the need for chemotherapy. However, 10–20 % of neonates with TAM and silent TAM subsequently develop ML-DS in the first 5 years of life when persistent *GATA1* mutant cells acquire additional oncogenic mutations, most often in cohesin or epigenetic regulator genes [12••, 13]. This review article discusses the recent clinical and biological advances in TAM and ML-DS and how these may impact on clinical management.

### Cellular and Molecular Pathogenesis of TAM and ML-DS

**Fig. 1:**
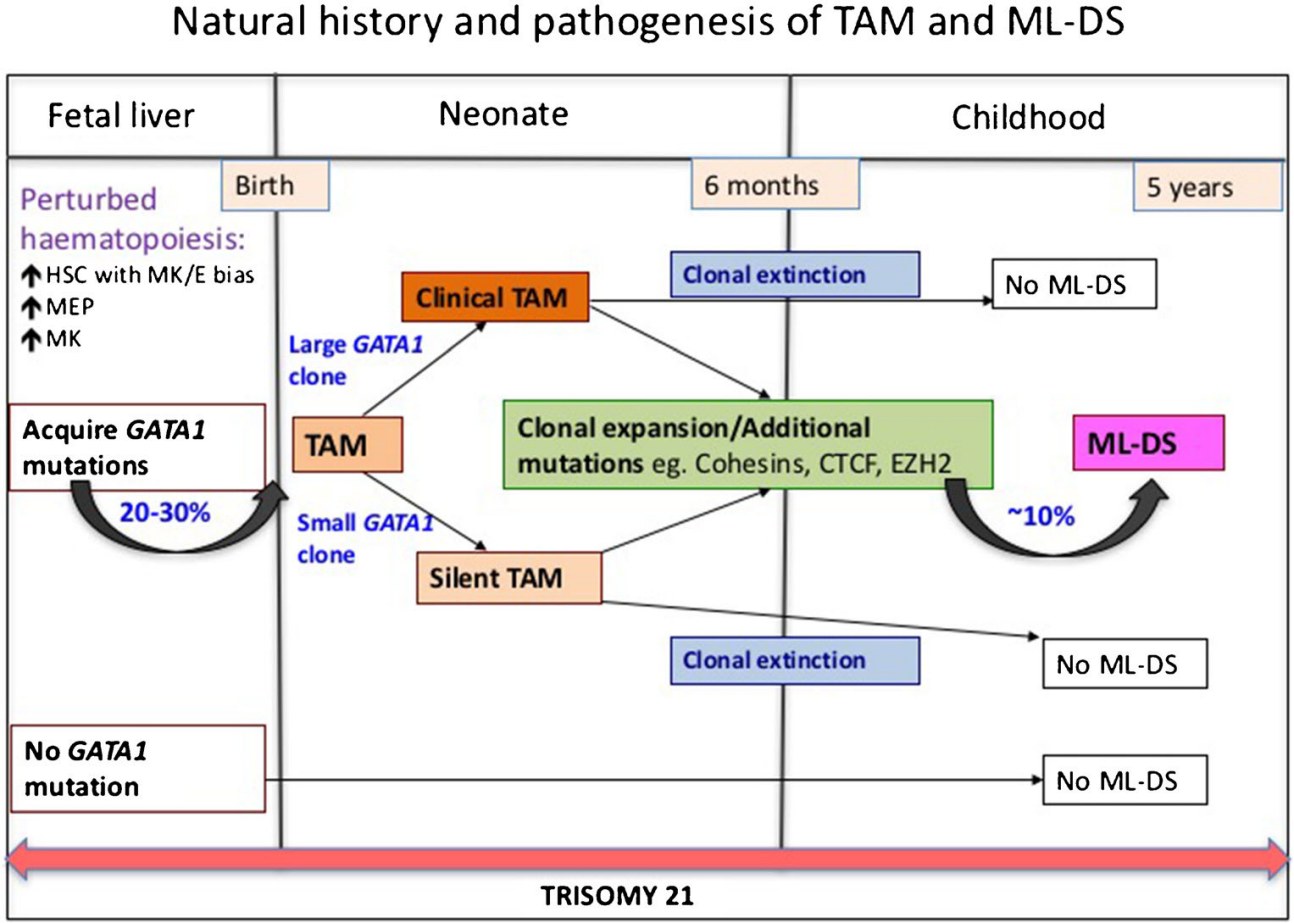
Natural history and pathogenesis of TAM and ML-DS. Schematic representation of molecular, biological and clinical data, indicating that transient abnormal myelopoiesis (*TAM*) and myeloid leukaemia of Down syndrome (*ML-DS*) are initiated before birth when fetal liver haematopoietic stem and progenitor cells (*HSPC*) trisomic for chromosome 21 demonstrate perturbed haematopoiesis with an expansion of megakaryocyte-erythroid progenitors (*MEP*) and megakaryocytes. These cells subsequently acquire N-terminal truncating *GATA1* mutations resulting in TAM in late fetal or early neonatal life. Although most cases of TAM spontaneously and permanently remit (∼90 %) by the age of 6 months, in ∼10 % of cases, additional genetic/epigenetic events lead to further clonal expansion resulting in ML-DS before the age of 5 years

The cellular and molecular events involved in initiation and evolution of TAM and ML-DS can best be understood as a three-step model which requires the presence within a fetal liver-derived haematopoietic stem or progenitor cell of (i) trisomy 21, (ii) an acquired *GATA1* mutation, and (iii) at least one additional oncogenic mutation (Fig. 1).

(i) Perturbation of fetal haematopoiesis by trisomy 21
  The initial event in trisomy 21-associated preleukaemic and leukaemic conditions is the perturbation of fetal haematopoiesis by trisomy 21 itself. It is known that by late in the first trimester of fetal life, haematopoiesis in the liver is abnormal in fetuses with trisomy 21 and that these changes precede the acquisition of *GATA1* mutations [14, 15••]. Specifically, trisomy 21 causes an increase in the numbers of megakaryocyte-erythroid progenitors (MEP) and an increase in the size and characteristics of the immunophenotypic haematopoietic stem cell (HSC) compartment [15••]. HSC and multipotent myeloid progenitors in trisomy 21 fetal liver proliferate more in vitro compared with normal fetal liver at the same stage of development and have increased erythroid-megakaryocyte output and gene expression [15••]. Despite the increase in megakaryocytes (MK) in trisomy 21 fetal liver, MK differentiation is impaired and platelet counts are reduced both in fetal blood and in neonates with Down syndrome suggesting that trisomy 21 itself causes dysmegakaryopoiesis [11••, 14, 15••].
  The molecular basis for these dramatic changes in fetal erythro-megakaryopoiesis is not yet clear. Some, but not all, of the features can be recapitulated either in elegant animal models [16–20] or in studies in human embryonic stem cells (ESC) and induced pluripotent stem cells (iPSC) [21, 22]. Together, these studies have implicated increased expression of various genes on chromosome 21, in particular *ERG* and *DYRK1a*, as important mediators of the abnormal megakaryopoiesis, although this does not seem to be sufficient to cause leukaemia in trisomic or disomic mouse models even when co-expressed with an N-terminally truncated *GATA1* gene. Interestingly, recent data using a panel of iPSC lines, suggest that trisomy of *RUNX1*, *ETS2*, and *ERG* might be sufficient, in combination with mutant *GATA1*, to explain many of the haematopoietic abnormalities seen in primary human fetal liver and TAM cells. Despite these interesting findings, it is now clear that trisomy 21 causes genome-wide changes in gene expression directly or indirectly affecting multiple genes on most chromosomes [23].
(ii) N-terminal truncating GATA1 mutations in TAM and ML-DS
  The link between acquired mutations in the *GATA1* gene and ML-DS was first identified more than 12 years ago in John Crispino’s lab [5] and rapidly followed by studies from a number of groups confirming the link with trisomy 21 as well as showing the same N-terminal truncating mutations in TAM [6–10]. The *GATA1* mutations that can be detected in all cases disappear when TAM (or ML-DS) enter remission indicating that these are acquired events [9, 12••]. Application of highly sensitive next-generation sequencing (NGS)-based methodology has recently shown that *GATA1* mutations are present in all cases of TAM or ML-DS and that they are present in 25–30 % of all neonates with Down syndrome [11••]. This means that *GATA1* mutations are necessary for the development of TAM/ML-DS, that they are acquired prior to birth in fetal cells and that they occur at an astonishingly high frequency. It seems likely that acquisition of such mutations confers a selective growth advantage to these cells during fetal life. The presence of multiple *GATA1* mutant clones in up to 25 % of neonates with Down syndrome is consistent with this [9, 11••]; however, the reason for their high frequency in Down syndrome remains unknown (these mutations are not found in normal, disomic cord blood) [9, 11••].
  The vast majority of acquired *GATA1* mutations (∼97 %) are found in exon 2 and the remainder in exon 3.1 of the *GATA1* gene, including insertions, deletions and point mutations [10]. These mutations lead to expression of a truncated GATA1s protein [5, 6] and the type of *GATA1* mutation does not predict which patients with TAM will later progress to ML-DS [10]. Since the *GATA1* gene is on the X chromosome, haematopoietic cells harbouring *GATA1* mutations express only GATA1s and no longer have the ability to produce the full-length GATA1 protein [5]. The main physiological role of GATA1 is as a regulator of normal megakaryocyte and erythroid differentiation [24]. How GATA1 transforms trisomy 21 fetal haematopoietic cells is unclear. Forced expression of GATA1s in fetal liver haematopoietic progenitors from GATA1 wild-type mice causes marked expansion of megakaryoblastic progenitors, supporting a gain of function mechanism [25, 26] and interestingly, Banno et al. using a trisomy 21 iPSC model also found that an increased level of expression of GATA1s might be responsible for the aberrant megakaryopoiesis they observed [27]. However, whether this is the main mechanism in trisomy 21 human cells and how GATA1s might transform trisomy 21 fetal haematopoietic cells remains an interesting question.
(iii) Mutational landscape of ML-DS
  The presence of an N-terminal truncating mutation in *GATA1* is necessary, but insufficient, for development of ML-DS. Recent whole genome and whole exome sequencing studies of ML-DS provide insight into the additional genetic events which co-operate with *GATA1* mutations and trisomy 21 to further transform haematopoietic cells from a usually transient preleukaemic syndrome (TAM) to an acute leukaemia (ML-DS), which is inexorably fatal unless eradicated with chemotherapy [12••, 13]. These show a high frequency of mutations (∼50 %) in all the key cohesin component genes (*RAD21*, *STAG2*, *SMC3* and *SMC1A*), as well as in *CTCF* (∼20 %) and in epigenetic regulators such as *EZH2* and *KANSL1* (45 %) [12••]. These genes encode proteins important for transcription regulation and long-range interactions that may be particularly vulnerable to disruption in trisomic cells. A smaller proportion of patients had mutation in RAS pathway genes (*NRAS*, *KRAS*, *CBL*, *PTPN11* and *NF1*) [12••, 13] that are also seen at high frequency in other childhood leukaemias, such as juvenile myelomonocytic leukaemia [28].
(iv) Role of the haematopoietic microenvironment
  Although the data from primary human tissues as well as ESC and iPSC, indicate that trisomy 21 causes cell intrinsic changes in fetal haematopoietic stem and progenitor cells, the fetal liver haematopoietic microenvironment may also contribute both to these changes and to expansion and/or maintenance of the mutant *GATA1* clone in TAM. Indeed, the natural history and clinical features of TAM clearly show this to be a fetal liver disease (see below). The nature of the growth factors that might mediate abnormal fetal haematopoiesis in TAM is unclear, although differences in the expression or responsiveness to the developmentally regulated IGF signalling pathway remain an attractive candidate [29].

### TAM: *Clinical Features*

TAM has a very variable clinical presentation: at one end of the spectrum, it may be detected as an incidental finding on review of a blood film in an otherwise well baby (10–25 % neonates) and at the other end of the spectrum, neonates with TAM may be very sick with disseminated leukaemic infiltration (10–20 % of neonates) presenting with massive hepatosplenomegaly, effusions, co-agulopathy and multiorgan failure [30–32, 33••]. The majority of neonates with clinical TAM (i.e. blasts >10 %) will have one or more of the well recognised clinical features of TAM which are summarised in Table 1. Amongst these features, hepatomegaly, splenomegaly, pericardial/pleural effusions and skin rash are seen more frequently in neonates with TAM compared with neonates without any *GATA1* mutations. Jaundice, on the other hand, is common in neonates with Down syndrome with or without TAM [11••, 30–32]. Importantly, however, as no single clinical feature is specific for TAM, it is essential to review the blood film of all neonates with Down syndrome to avoid missing cases of TAM and to assess the significance of the clinical features shown in Table 1 [11••]. This is also important in the setting of delayed onset or prolonged hyperbilirubinaemia in neonates with Down syndrome as this may be the presenting feature of progressive TAM-associated liver fibrosis that may be fatal. Although the majority of cases of TAM present within the first few days of life, TAM may also present in fetal life either with hydrops fetalis or with features similar to those presenting postnatally [34•].

**Table 1:** Clinical and haematological features of neonates with Down syndrome with and without *GATA1* mutations

Clinical feature (% neonates)	TAM^a^	Silent TAM^b^	Down syndrome (no GATA1 mutations)
Hepatomegaly	40	<5	4
Splenomegaly	30	<1	<1
Skin rash	11	<1	<1
Pericardial/pleural effusion	9	<1	<1
Jaundice	70	60	50–60
Abnormal LFTs	25	<10	<10
Abnormal coagulation	10–25	∼5	∼5
Thrombocytopenia (<100 × 10^9^/l)	50	50	50
Leucocytosis (>26 × 10^9^/l)	∼50	10	10–15
Anaemia (<130 g/L)	5–10	<5	1–5

Data based on information from refs. [11••, 30–32, 33••]

^a^Peripheral blood blasts >10 % and one or more acquired *GATA1* mutations

^b^Silent TAM: Peripheral blood blasts ≤10 % and one or more acquired *GATA1* mutations

### TAM: Laboratory Features

TAM causes several haematological abnormalities. Characteristically, the main features are leucocytosis and increased peripheral blood blasts. Leucocytosis is present in 30–50 % of cases of TAM and typically includes increased neutrophils, myelocytes, monocytes and basophils [11••, 31, 33••]. The platelet count may be elevated, normal or reduced, and thrombocytopenia is not more common in neonates with TAM than in DS neonates without TAM [11••]. Similarly, although the median haemoglobin is lower in neonates with TAM compared with neonates with Down syndrome without TAM, anaemia is uncommon [11••, 31, 33••]. A deranged coagulation profile is reported to occur in 20–25 % of cases, although disseminated intravascular co-agulopathy (DIC) is usually confined to cases where there is severe liver dysfunction due to hepatic infiltration by blast cells [30–32, 33••]. Hepatic dysfunction is manifested by severe conjugated hyperbilirubinaemia and often, but not always, accompanied by elevated transaminases [30–32, 33••, 39]. The only one of these laboratory features that is specific for a diagnosis of TAM is a high number of circulating blast cells. However, one of the most challenging aspects of diagnosis of TAM has been establishing whether or not there is a threshold value for the percentage of blasts that is reliable for diagnosis in the absence of molecular confirmation by *GATA1* mutation analysis.

#### Blast count, morphology and immunophenotype

Although TAM is characterised by increased peripheral blood blasts, it is now known that blast cells are seen on the blood film of almost all neonates (∼98 %) with Down syndrome and may account for 15–20 % of the circulating leucocytes in neonates shown to have no *GATA1* mutations [11••]. There is no internationally agreed definition of a percentage blast threshold that constitutes ‘increased peripheral blood blast cells’. In the Oxford Imperial Down Syndrome Cohort (OIDSC) Study, we addressed this question by prospectively classifying cases with blasts of >10 % and a *GATA1* mutation in the first 14 days of life as TAM. In the preliminary analysis of the first 200 neonates with Down syndrome recruited into the study, 17 (8.5 %) fulfilled these criteria for a diagnosis and these criteria identified all neonates with clinical features of TAM, including all with severe disease [11••]. This analysis also showed that ∼25 % of neonates with blasts >10 % do not have a *GATA1* mutation even when very sensitive NGS-based methods are used. On the other hand, 18/70 neonates in the OIDSC study (26 %) neonates with blasts ≤10 % (range 1–10 %) had a *GATA1* mutation when NGS-based methods were used; these cases had no clinical and haematological features suggestive of TAM and were designated ‘silent TAM’. Taken together these data indicate that an accurate diagnosis of TAM relies on both the presence of blasts and a *GATA1* mutation and suggest that a blast threshold of >10 % will identify all neonates with Down syndrome with TAM who may require chemotherapy and close monitoring during the neonatal period. However, this blast threshold is not specific for TAM and is not sufficiently sensitive to identify the majority of neonates who have *GATA1* mutations. Typically, the blast cells in TAM are described as megakaryoblastic with cytoplasmic blebbing and basophilic cytoplasm; however, in our experience the morphology of the blasts is highly variable. Similarly, the immunophenotype of the blast cells is highly variable; the characteristic pattern of co-expression of stem cell markers (CD34 and CD117), myeloid markers (CD33/CD13), platelet glycoproteins (CD36, CD42, CD61) together with CD56 and CD7 is heterogeneous both within and between cases [35–38]. At present, there is no distinguishing morphological and immunophenotypic profile that can accurately discriminate TAM from cases where there are no *GATA1* mutations [11••].

#### Silent TAM

As mentioned above, at least half of all Down syndrome neonates with *GATA1* mutations have a peripheral blood blast percentage of 1–10 % and have no clinical features associated with TAM (Table 1) [11••]. This is an important group of neonates because the presence of the *GATA1* mutation means that they are at risk of subsequently developing ML-DS if the mutant *GATA1* clone persists [11••]. It is likely that the reason for the lack of clinical features is the small size of the mutant *GATA1* clone at birth since the OIDSC study showed a strong correlation between the size of the mutant clone and the percentage of peripheral blood blasts [11••].

### TAM and Silent TAM: *GATA1* Mutation Analysis

The recognition of silent TAM means that detection of *GATA1* mutations for clinical diagnosis requires sensitive as well as specific and reliable methods. Current available methodologies for *GATA1* mutation analysis are direct Sanger sequencing (sensitivity 10–30 %), dHPLC (sensitivity 2–10 %) and various methods of NGS (sensitivity 0.3–2 %) [9, 10, 11••, 12••]. Each method has technical limitations, advantages and disadvantages, but only NGS-based methods are sufficiently sensitive for initial diagnosis as neither direct Sanger sequencing nor dHPLC are able to reliably detect small *GATA1* mutant clones (<10 %) that are of clinical significance [11••]. The value of monitoring mutant *GATA1* clones after diagnosis is currently not clear; this would require an extremely sensitive method and an important limitation is that neonates with TAM may have more than one *GATA1* mutant clone and that ML-DS may develop from either or both major and minor *GATA1* clones present at birth [11••, 12••].

### Natural History of TAM and Progression to ML-DS

Most neonates with TAM (>80 %) undergo spontaneous resolution of both clinical and laboratory abnormalities within 3 months after birth with a 5-year overall survival of ∼80 % and event-free survival of ∼60 % [30–32, 33••]. Complete remission is often characterised first by normalisation of blood counts and disappearance of peripheral blasts followed by resolution of clinical symptoms such as hepatomegaly [39]. The overall mortality is reported to be ∼20 %, however, only half of the deaths are directly attributable to TAM usually due to hepatic failure secondary to fibrosis and blast cell infiltration [30–32, 33••].

Estimates of the risk of progression of TAM to ML-DS are mainly based on retrospective studies and suggest that 20–30 % of neonates with TAM will subsequently present with ML-DS [30–32, 33••]. Since it is now known that the frequency of GATA1 mutations at birth is much higher than previously realised (25–30 % of all neonates with Down syndrome) and since population-based estimates of the frequency of ML-DS indicate that ∼1.5 % of children with Down syndrome will develop this leukaemia before the age of 5 years [1], this suggests that the risk of progression is lower than these original estimates (around 5–10 %) given that silent TAM also has the potential to transform to ML-DS. In some cases, there is overt progression/evolution of TAM to ML-DS with persistent abnormal haematology and an indolent myelodysplastic syndrome; in other cases, there is a variable apparent remission before development of ML-DS [11••, 30, 31]. *GATA1* mutations are detected in all cases of ML-DS [11••, 12••] and are therefore essential for progression to ML-DS. Factors, which reliably predict transformation of TAM to ML-DS, have not been identified yet. The type of *GATA1* mutation does not seem to play a role [10]. Data on the size of the *GATA1* mutant clone at birth as a predictor of later ML-DS are too preliminary at present. The only clinical factor shown in multivariate analysis to predict transformation of TAM to ML-DS is the presence of pleural effusion in the neonatal period [31].

### TAM: Management

Most neonates with TAM undergo spontaneous resolution and do not need treatment. However, neonates with progressive life-threatening symptoms such as *hydrops fetalis*, extreme leucocytosis (WBC >100 × 10^9^/l), hepatopathy, DIC with bleeding, renal and/or cardiac failure may benefit from chemotherapy as the mortality rate may be up to 20 % [30–32, 33••, 39]. A summary of the outcome of treatment from these studies is shown in Table 2. As TAM blasts appear to be very sensitive to cytarabine [40] and early observational studies showed promising results with very low doses of cytarabine [41], currently used regimens are based on this approach. The Berlin-Frankfurt-Münster group recommended treatment with cytarabine (0.5–1.5 mg/kg for 3–12 days) for neonates with TAM and clinical impairment due to thrombocytopenia, signs of cholestasis or liver dysfunction or high white cell count (>50 × 10^9^/l) [31]. Out of 146 patients, 28 received treatment with cytarabine many of which had hepatic fibrosis and required intensive support. Survival in the treated and untreated groups was very similar (5-year overall survival 78 ± 8 vs. 85 ± 3 %, *p* = 0.44), suggesting that treatment might have been beneficial given that the treated neonates had much more severe disease [31]. The Children’s Oncology Group identified 38 of 135 patients as having life-threatening symptoms and 24 received cytarabine, given as a continuous infusion at a dose of 3.33 mg kg^−^ /day^−1^ for 7 days. The survival rate for the treatment group was disappointing (51 %) most likely reflecting both the severity of the disease and the high rate of haematological toxicity (96 % grade 3/4 myelosuppression) perhaps because of the higher dose and continuous infusion regimen [33••]. More recently, a preliminary report from Muramatsu et al. of a large study in neonates with TAM reported a significant improvement in 1 year survival when neonates with extreme leucocytosis (>100 × 10^9^/l) were treated with cytarabine [52]. However, there is no evidence at present that treatment with cytarabine has a significant impact on the likelihood of disease progression to ML-DS [31, 33••].

**Table 2:** Mortality and transformation to myeloid leukaemia of Down syndrome (ML-DS) in neonates with Down syndrome and transient abnormal myelopoiesis (TAM)*

No. of patients	Massey [30]	Klusmann [31]	Muramatsu [32]	Gamis [33••]	Total
	47	146	70	135	398
Early death	8 (17 %)	22 (15 %)	16 (23 %)	29 (21 %)	75 (19 %)
TAM-associated hepatic failure	8 (17 %)	7 (5 %)	11 (16 %)	13 (10 %)	39 (10 %)
Other TAM deaths	0	6 (4 %)	4 (5.7 %)	1 (0.7 %)	11 (3 %)
Non-TAM deaths	0	9 (6 %)	1 (1.4 %)	15 (11 %)	25 (6 %)
ML-DS (of survivors)	9 (23 %)	29 (23 %)	12 (22 %)	21 (20 %)	71 (22 %)

* Clinically diagnosed TAM. Data based on information from refs. [30–32, 33••]

### ML-DS: Clinical Features

ML-DS is classified as a specific subtype of AML in the World Health Organisation (WHO) classification [42]. This leukaemia is unique to Down syndrome and has several distinct features. Firstly, ML-DS presents at a median age of 1–1.8 years and is rare after the age of 4 years [43, 44]. Secondly, most cases of ML-DS have a clinical history consistent with preceding TAM in the neonatal period, and for those that have no such history, the most likely reason is the absence of appropriate diagnostic tests at birth [11••]. Consistent with this, *GATA1* mutations are found on neonatal bloodspots from neonates with ML-DS even in the absence of an antecedent history of TAM [9]. ML-DS often shows an indolent presentation with myelodysplasia and progressive pancytopenia, in particular thrombocytopenia and leucopenia, with a low percentage of circulating blasts for many months before the development of ML-DS [43, 45, 46]. Since the circulating blast count is often low in ML-DS and the predominant haematological picture may be of slowly progressive pancytopenia, a bone marrow aspirate is usually essential for the diagnosis of ML-DS. However, this is often associated with a ‘dry tap’ secondary to marked bone marrow fibrosis and a bone marrow trephine may be necessary to confirm ML-DS—it is not clear that the conventional bone marrow blast threshold used in acute myeloid leukaemia is of value in ML-DS in view of the natural history of the condition and the difficulty in obtaining a representative sample.

### ML-DS: Laboratory Features

Almost all patients with ML-DS have thrombocytopenia and most also have anaemia and neutropenia. In contrast to TAM, the leucocyte count is usually low. However, the blast cells are similar to those in TAM, with a typical megakaryoblastic morphology [43, 47] and co-expression of stem/progenitor cell markers (CD34, CD117), myeloid (CD33), megakaryocytic (CD42b and CD41) and erythroid markers (CD36 and glycophorin A) as well as CD7 [35, 37, 47]. ML-DS has a distinct cytogenetic profile compared with sporadic AML in children without Down syndrome in that the favourable cytogenetic changes such as *AML*-*ETO* t(8;21), *PML*-*RARA* t(15;17), *MLL* t(9;11) and *CBFB MYH11* inv[16] nor the acute megakaryoblastic leukaemia-associated translocations *RBM15*-*MKL1* t(1;22) and t(1;3) occur in ML-DS [48, 49]. Instead, several karyotypic abnormalities are more frequent in ML-DS than in children without Down syndrome, including trisomy 8, trisomy 11, trisomy 21, del (6q), del(7p), del(16q) and dup(1p) [49]. *GATA1* mutations are always present in ML-DS blasts and, where available, targeted or genome-wide next-generation sequencing usually reveals mutations in additional known oncogenes, such as the cohesin genes, as described above [12••, 13].

### ML-DS: *Treatment and Outcome*

A number of studies show that children with ML-DS have better outcomes compared with children without Down syndrome with long-term survival of 74–91 % (Nordic group 10-year OS 74 %, *n* = 61; the Children’s Cancer Group (CCG) 5–year OS 79 %, *n* = 161; BFM study group 3-year OS 91 %, *n* = 67 and the Medical Research Council 5-year OS, 75 %, *n* = 36) [33••, 43, 45, 50]. Early studies showed that treatment failure in children with Down syndrome due mainly to chemotherapy-related toxicity, particularly related to anthracylines, with a higher rate of induction deaths in ML-DS [45]. Most groups now use a reduced dose of anthracycline that is less toxic and appears to give similar efficacy [55–57]. The outlook for children with ML-DS who relapse is very poor. In a review of the Japanese data, Taga et al. reported a 3-year overall survival rate of 25.9 % [51], similar to the 21 % overall survival in the POG 9421 and CCG-22891 AML studies [52]. There appears to be no role for allogeneic stem cell transplant (SCT) in first-line therapy for ML-DS due to efficacy of chemotherapy and the ongoing high rate of toxicity associated with SCT. Even the role of SCT as salvage therapy is unclear. A recent review by the Center for International Blood and Marrow Transplant Research (CIBMTR) of outcome data from 28 children transplanted for ML-DS compared with 80 non-Down syndrome children with AML reported a 3-year probability of overall survival of only 19 % due to the high rate of relapse and transplant-related mortality [53]. The results of a small retrospective study in 15 patients suggest that transplant-related toxicity might be reduced by using reduced intensity conditioning regimens (*n* = 5; 80 % event-free survival) compared with standard myeloabalative regimens (*n* = 10; 10 % event-free survival) [54]; however, these results remain to be confirmed in larger studies.

## Conclusion

Children with Down syndrome have a markedly increased risk (∼150-fold) of developing acute myeloid leukaemia, known as ML-DS) compared with children without Down syndrome. ML-DS is preceded by a clonal neonatal preleukaemic disorder, known as TAM, which maybe clinically overt or silent. TAM and ML-DS have unique biological, cytogenetic and molecular characteristics. There are at least three distinct steps in the pathogenesis of ML-DS. First, trisomy 21 perturbs fetal haematopoiesis, providing the ideal cellular context for the second step: transformation of these fetal haematopoietic cells by acquired N-terminal truncating mutations in the *GATA1* gene to produce the clinical syndrome TAM. While the majority of cases of TAM resolve without sequlae as the *GATA1* mutation is lost, ∼10 % of children harbour residual *GATA1*-mutant cells which then, in the third step, acquire transforming mutations in additional oncogenes leading to ML-DS. Uncovering the mechanisms which underlie these events remains an exciting challenge and is at last beginning to offer real prospects of translation of these finding into useful therapeutic advances for children with Down syndrome so that we can improve treatment and outcome by investigating new agents that could potentially improve their leukaemia-free survival without additional toxicity.
